# Live-Cell Imaging of Early Steps of Single HIV-1 Infection

**DOI:** 10.3390/v10050275

**Published:** 2018-05-19

**Authors:** Ashwanth C. Francis, Gregory B. Melikyan

**Affiliations:** 1 Department of Pediatrics, Emory University, Atlanta, GA 30322, USA; ashwanth.francis@emory.edu; 2 Children’s Healthcare of Atlanta, Atlanta, GA 30322, USA

**Keywords:** Single HIV imaging, viral fusion, uncoating, maturation, nuclear import, infection

## Abstract

Live-cell imaging of single HIV-1 entry offers a unique opportunity to delineate the spatio-temporal regulation of infection. Novel virus labeling and imaging approaches enable the visualization of key steps of HIV-1 entry leading to nuclear import, integration into the host genome, and viral protein expression. Here, we discuss single virus imaging strategies, focusing on live-cell imaging of single virus fusion and productive uncoating that culminates in HIV-1 infection.

## 1. Introduction

The human immune deficiency virus type-I (HIV-1) enters target cells by membrane fusion mediated by interactions between the viral envelope glycoprotein gp120/gp41 with CD4 and chemokine receptors, CCR5 and CXCR4. HIV-1 fusion results in the cytoplasmic delivery of the conical core composed of ~1600 molecules of capsid protein (CA) that encloses two copies of positive-strand viral genomic RNA (vRNA), the viral enzymes reverse transcriptase (RT), and integrase (IN), as well as other viral and cellular proteins (reviewed in [1]). The released viral core is transported from the fusion sites towards the nucleus by CA-interacting cellular motor proteins [2,3,4,5,6]. Loss of CA from the viral core is referred to as uncoating [7,8,9]. Timely uncoating is linked to the synthesis of the viral cDNA (vDNA) from vRNA by RT. The resulting pre-integration complex (PIC) containing the newly formed vDNA and IN molecules is transported through interactions with essential host factors into the nucleus, where IN catalyzes stable insertion of vDNA into actively transcribing regions of chromatin [10,11,12]. Upon HIV-1 DNA integration, the cellular transcription and translation machinery produces the viral genomic RNA and structural proteins that assemble at the plasma membrane and form viral particles that exit the cell for a subsequent round of infection.

The HIV-1 life cycle has been traditionally studied using genetic, molecular biology, structural biology, and biochemical tools. The more recent development of live cell fluorescent probes, along with advancements in microscopic techniques, has opened new avenues for visualizing virus infection in living cells. Importantly, the ability to fluorescently tag different viral components has provided new insights into HIV-1 biology (reviewed in [13,14,15,16,17]). Live-cell-based virus imaging provided a better understanding of key steps of infection, including virus–cell fusion [18,19,20,21,22,23,24,25,26], cytoplasmic trafficking of HIV-1 cores [2,3,4,5,6], uncoating [4,27,28,29,30], virus nuclear import [28,31,32,33], transcription [34,35,36], virus assembly and budding [37,38,39,40,41,42,43,44,45,46] and cell–cell transmission [47,48,49,50].

Here, we will review new fluorescent labeling approaches, focusing on those developed in our laboratory, that enable robust live-cell visualization of single HIV-1 fusion and uncoating that result in productive infection.

## 2. A Bi-Functional Reporter of Single Virus Protease Activity and Virus–Cell Fusion

We and others have implemented real-time imaging of single virus-cell fusion by co-labeling HIV-1 particles with a fluid-phase and fiduciary markers [18,19,20,21,22,23,24,25,26,29]. Here, a fluid-phase marker is introduced into virions by inserting a green fluorescent protein (GFP)flanked by viral protease cleavage sites between the matrix (MA) and CA domains of the Gag polyprotein (referred to as Gag-internal-GFP or Gag-iGFP) [19,39]. During virus maturation, GFP molecules are released from Gag-iGFP by the viral protease and remain trapped inside the virion. Viral fusion is sensitively detected as a loss of this fluid-phase marker, and the inclusion of a fiduciary marker associated with the viral core or the viral membrane allows robust particle tracking before and after fusion. Labeling of the viral core can be accomplished by fluorescently tagging Vpr (viral protein R) or IN [2,31,51,52,53,54] or using the virion-packaged cellular proteins, such as apolipoprotein B mRNA editing enzyme, catalytic polypeptide-like APOBEC-3F (A3F) [33]. The HIV-1 membrane has been labeled by incorporating lipophilic dyes [2,19] or fluorescently labeled transmembrane proteins [23], attaching quantum dots [55], or by incorporating unnatural sugars into the viral surface glycoproteins followed by click-labeling with an organic dye [20].

**Figure 1:**
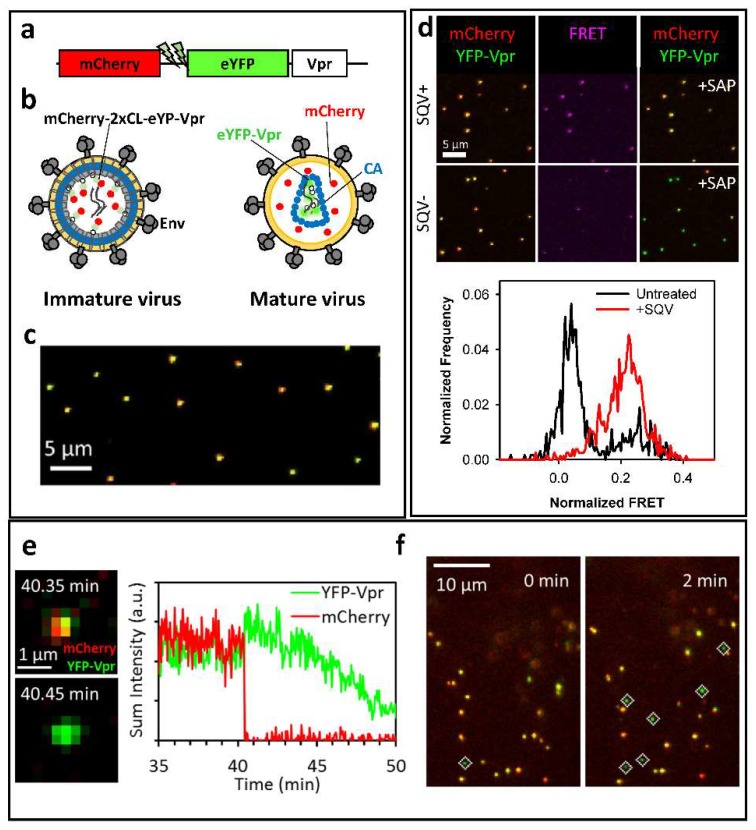
Bi-functional fluorescence marker for HIV-1 protease activity and fusion. (**a**) Illustration of the bi-functional mCherry-2xCL-eYFP-Vpr marker; (**b**) Cartoon of immature and mature HIV-1 particles labeled with mCherry-2xCL-eYFP-Vpr. eYFP fluorescence is quenched in immature viral particles as a result of FRET between the eYFP and mCherry. eYFP fluorescence is enhanced in mature virions following the release of mCherry from the bi-functional marker; (**c**) A representative image of single viral particles bound to a cover glass showing a nearly perfect colocalization of mCherry and eYFP; (**d**) Immature viruses produced in the presence of saquinavir (SQV) exhibit high Forster Resonance Energy Transfer (FRET) between eYFP and mCherry (middle panel). Corresponding fluorescence images of single virions before (left) and after (right) saponin lysis (+SAP) are shown; (**e**) Single virus fusion is detected based upon a quick release of fluid-phase mCherry marker from eYFP-Vpr labeled mature HIV-1 core. The fluorescence intensity traces of the fusing particle are shown on the right; (**f**) Automated detection of post-fusion HIV-1 cores in CV1 cells as eYFP-Vpr-labeled labeled puncta negative for mCherry (marked by white diamonds). Data are adapted from Sood et al., 2017.

The above virus co-labeling techniques require cell transfection with multiple plasmids, including those encoding for different fluorescent constructs. A major disadvantage of these approaches is the relatively poor HIV-1 co-labeling efficiency, which tends to vary between viral preparations, and reduced infectivity [22]. In order to overcome these limitations, we have recently developed a dual-color fluorescent marker that is incorporated into viral particles through the Vpr protein. This marker, referred to as mCherry-2xCL-eYFP-Vpr, contains a monomeric Cherry/enhanced yellow fluorescent protein (mCherry/eYFP) tandem linked to the N-terminus of HIV-1 Vpr (Figure 1a) [22]. A short sequence containing a tandem HIV-1 protease cleavage site (2xCL) links the two fluorescent proteins. Upon HIV-1 protease activation, this linker is cleaved, ensuring the release of mCherry from eYFP-Vpr, which remains associated with the viral core (Figure 1b). The produced free mCherry molecules serve as a fluid-phase marker for sensitive detection of single virus fusion. Labeling of HIV-1 particles with mCherry-2xCL-eYFP-Vpr results in a nearly perfect colocalization of the two markers incorporated into the virions at 1:1 ratio (Figure 1c), without compromising the virus infectivity.

An important advantage of the 1:1 ratio of mCherry and eYFP molecules in virions containing mCherry-2xCL-eYFP-Vpr is the ability to assess the viral protease activity in single particles [22]. The close proximity of mCherry and eYFP in the uncleaved bi-functional construct results in efficient Forster Resonance Energy Transfer (FRET) between eYFP and mCherry. Thus, FRET measurements or simply the measurements of the relative intensities of eYFP (donor) and mCherry (acceptor) enable the discrimination between immature (high FRET, low eYFP signal) and mature (low FRET, high eYFP signal) viruses (Figure 1d). Further support for the FRET-based prediction of the virus maturation status is provided by an excellent correlation between low FRET and release of mCherry from virions upon saponin treatment (Figure 1d).

Importantly, the fixed ratio of eYFP and mCherry molecules in virions allows streamlining the detection of viral fusion, using software that automatically detect changes in “color-balance” (the normalized difference between yellow and red fluorescence) of individual particles in live cells. Here, post-fusion particles are readily detected by identifying eYFP-Vpr puncta that have very low or no mCherry signal (Figure 1e,f). However, quantifying post-fusion cores in the cytoplasm over time is challenging due to the loss of eYFP-Vpr signal within 20–30 min after the viral content release in HeLa- and CV1-derived cell lines [22,24]. This problem can be alleviated through the use of proteasome inhibitors that minimize the loss of eYFP-Vpr puncta and allow reliable quantification of post-fusion HIV-1 cores, following the quick and efficient fusion mediated by the Vesicular Stomatitis Virus G (VSV-G) or Avian Sarcoma and Leukosis Virus (ASLV) Env glycoproteins [22]. Importantly, this analysis can be carried out after fixing cells at varied times post-infection, without real-time single particle tracking.

## 3. Labeling CA to Study Single HIV-1 Uncoating

Disassembly of the HIV-1 core after delivery into the cytoplasm, which is commonly referred to as uncoating, is arguably the least understood step of virus infection. Whereas CA is the main determinant for the cytoplasmic transport, nuclear import, and integration site selection (reviewed in [8,9,12,56]), a partial or complete disassembly of the conical capsid shell is thought to be required for HIV-1 nuclear entry. Several excellent studies, using biochemistry, molecular/cell biology, and fixed-cell-based microscopy tools, have contributed to our understanding of the uncoating process (reviewed in [8,9]). These studies led to the realization that the timing and localization of uncoating are key to understanding the multiple roles of CA in HIV-1 entry. Live cell imaging of single HIV-1 uncoating can uniquely reveal the spatio-temporal regulation of this process during viral infection. However, the development of live-cell imaging assays to visualize single HIV-1 uncoating has been limited, primarily due to difficulties associated with fluorescent labeling of CA without compromising the virus infectivity [30,57,58]. For instance, tagging the CA protein with a small tetracysteine (TC) motif that can be labeled with bi-arsenical fluorescent dyes, compromises virus infectivity [30,57,58]. This defect could be partially rescued by mixing labeled and unlabeled Gag proteins [29,30,57].

**Figure 2:**
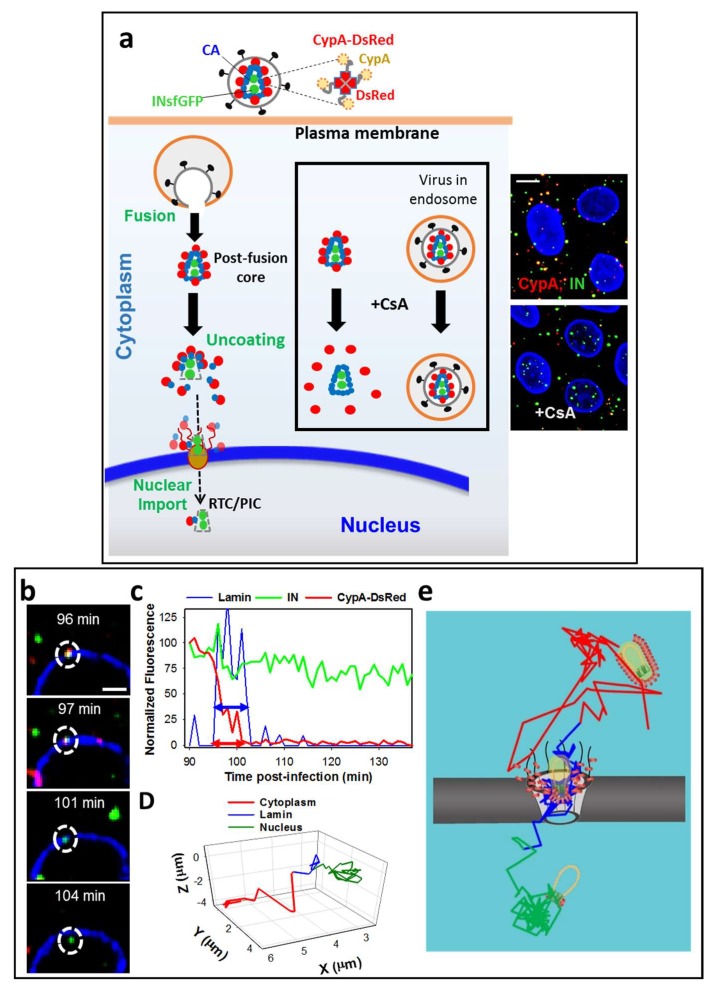
A novel capsid protein (CA) marker CypA-DsRed enables visualization of single HIV-1 uncoating. (**a**) Illustration of HIV-1 co-labeling with INsfGFP that marks the viral pre-integration complexes and the tetrameric CA marker, CypA-DsRed (inset), that remains bound to CA after viral fusion. Single HIV-1 uncoating and nuclear import are shown. The second advantage of this labeling strategy (shown boxed) is the ability to identify post-fusion cores by Cyclosporine A (CsA) treatment. CsA selectively displaces CypA-DsRed from post-fusion cores, but not from intact viruses trapped in endosomes; (**b**–**e**) Uncoating and nuclear import of single HIV-1 cores in TZM-bl cells. Confocal images (**b**) and fluorescence intensity traces (**c**) of uncoating and nuclear import are shown; (**d**) Single particle trajectory corrected for the nucleus movement. Segments of the trajectory are colored to mark the cytoplasmic transport, docking and intra-nuclear movement. Dotted circles in (**b**) mark a single viral complex uncoating and entering the nucleus. Double arrows in (**c**) illustrate the virus docking time (colocalization with the lamin signal, blue) and the time of uncoating after docking (red). A model for HIV-1 uncoating and nuclear import is overlaid onto an actual 2D-trajectory of single HIV-1 undergoing uncoating and nuclear import (**e**). Scale bar 5 µm in (**a**) and 2 µm in (**b**). Adapted from Francis et al., 2016 and Francis and Melikyan, 2018.

To circumvent the disruptive effects of direct CA labeling, we have recently designed a novel cyclophilin A (CypA) based marker, where the carboxy-terminal domain of CypA is fused to the *Discosoma* sp. Red fluorescent protein (DsRed), to generate CypA-DsRed [27]. Like CypA, CypA-DsRed naturally incorporates into virions by specifically binding to the residues G89 and P90 within the CypA binding loop of CA [59,60,61]. However, unlike the CypA fusions with monomeric fluorescent proteins [57], the tetrameric CypA-DsRed efficiently incorporates into virions (Figure 2a) as a result of the increased binding avidity [62]. Importantly, the high-avidity binding of CypA-DsRed to Gag/CA in virus-producing cells does not considerably affect HIV-1 infectivity in TZM-bl or Jurkat cells. Perhaps owing to a sub-stoichiometric binding to Gag/CA [27,28], CypA-DsRed incorporation into HIV-1 does not perturb the CA interactions with the target cell restriction factors, TRIMCyp [63,64], and the cytosolic CPSF6-358 fragment [65]. Also, overexpression of this CA marker in target TZM-bl or 293T cells does not affect infection of unlabeled viruses [27]. Notably, the ability of virus-incorporated CypA-DsRed to rescue HIV-1 infection of target CypA-null Jurkat cells [66] implies that CypA-DsRed remains tightly bound to CA during viral infection and thereby functionally compensates for the lack of CypA in Jurkat cells. Through the high-avidity non-disruptive binding to the HIV-1 core, CypA-DsRed appears to faithfully report the loss of CA from the post-fusion cores co-labeled with integrase-super-folderGFP (INsfGFP) [27] (Figure 2a). This notion is supported by the concomitant loss of CA documented by immunolabeling for CA/p24. As expected, the loss of CypA-DsRed is modulated, both in vitro and in living cells (see below and [27]), by the CA mutations that alter the HIV-1 core stability [67].

Another important advantage of the CypA-DsRed-based CA labeling strategy is the ability to discriminate between double-labeled virions trapped in endosomes and post-fusion HIV-1 cores, which has been a challenge for other virus co-labeling strategies (e.g., [2,68]). The CypA-binding drug Cyclosporin A (CsA) displaces the CypA-DsRed marker from post-fusion HIV-1 cores that retain CA. Through the use of CsA, we have unambiguously identified post-fusion cores in the cytoplasm that have not completed uncoating and quantified their CA content at different times after infection [27] (Figure 2a, boxed inset).

## 4. Single HIV-1 Uncoating In Vitro

We have recently developed an in vitro HIV-1 uncoating assay that takes advantage of the CypA-DsRed CA marker [27]. Single viral particles co-labeled with INsfGFP and CypA-DsRed are bound to a cover glass, permeabilized with a detergent or saponin, and fixed with paraformaldehyde at varied time-points after permeabilization. To visualize CA, the permeabilized particles are immuno-stained with anti-p24 antibodies. Uncoating is measured by assessing the loss of the CA/p24 and CypA-DsRed signals from the INsfGFP-labeled viral complexes [27]. This approach allows one to readily visualize all viral complexes irrespective of their CypA-DsRed/CA content and determine the fraction of uncoated complexes as a function of time. The identical kinetics of CypA-DsRed and CA/p24 loss from INsfGFP complexes strongly support the utility of the new CA marker for visualizing the HIV-1 uncoating in vitro. As expected, the kinetics of CA/p24 and CypA-DsRed loss after permeabilization is markedly modulated by changes in the capsid stability, with immediate uncoating of viruses made of the unstable (K203A) CA mutant and delayed uncoating of the hyper-stable (E45A) mutant, described in [67]. Importantly, similar to other reports [69], the addition of cytosolic extracts stabilizes the HIV-1 cores in vitro, as evidenced by their delayed uncoating [27]. Collectively, these results imply that the loss of CypA-DsRed from INsfGFP labeled complexes is a reliable indicator of single virus uncoating.

## 5. Time-Resolved Imaging of Single HIV-1 Uncoating in the Cytoplasm

We have used the CypA-DsRed marker in combination with INsfGFP labeling to visualize single HIV-1 uncoating in TZM-bl cells. INsfGFP remains associated with RTC/PICs after uncoating [32,51,70,71] and enables tracking single viral complexes before and after the loss of CypA-DsRed [28]. Fluorescently labeled HIV-1 is pseudotyped with the Vesicular Stomatitis Virus G glycoprotein (VSV-G) to allow efficient fusion and delivery of viral cores into the cytoplasm. Synchronized viral fusion is achieved by binding the viral particles to cells at 4 °C, shifting to 37 °C and immediately imaging the cells on a confocal microscope stage maintained at 37 °C under 5% CO_2_. Live-cell imaging of the entire cell volume is performed at a relatively high temporal resolution (typically, every 20–30 s) for a 2-h window. This imaging regime allows tracking of single INsfGFP complexes and reveals the time-course of HIV-1 uncoating, as determined by the loss of CypA-DsRed. We define uncoating as a terminal loss of CypA-DsRed to the background or near background level, without a subsequent release of the remainder of this marker.

Two distinct uncoating phenotypes have been observed using the above labeling scheme. The overwhelming majority (~95%) of the cores uncoat within 90 min post-infection, as evidenced by an abrupt (within ~2 min) loss of CypA-DsRed. Addition of CsA that displaces CypA-DsRed from the cytoplasmic cores, but not from intact viral particles trapped in endosomes, at this time point reveals a minor fraction (~5%) of cores that retain the CA marker. Tracking of these single INsfGFP puncta showed a gradual loss of CypA-DsRed from the rare long-lived cores over several hours in the cytoplasm and in the vicinity of the nucleus [27]. For these cores, uncoating could be initiated shortly after viral fusion, but completed at the nuclear membrane.

Although the overwhelming majority of the HIV-1 cores lose CypA-DsRed/CA at early times after infection, these events do not appear to represent an infectious pathway. Most INsfGFP complexes disappear in the cytoplasm within ~30 min after uncoating and the loss of single IN complexes could be prevented by treating the cells with proteasome inhibitors, MG132 or lactacystin [28]. Notably, treatment with proteasome inhibitors increases the virus infectivity and, proportionally, the nuclear import of INsfGFP complexes. This finding implies that post-uncoating IN complexes are degraded by proteasomes and that HIV-1 uncoating that culminates in infection occurs at the late stages of virus entry, perhaps at the nuclear pore.

## 6. Docking at the Nuclear Pore

The nuclear compartment has been labeled with fluorescently tagged histone proteins or DNA-binding dyes, such as DAPI. However, this labeling strategy does not clearly define the nuclear membrane. In order to reliably track single HIV-1 docking at the nuclear pore and nuclear import, it is essential to clearly visualize the nuclear membrane. This has been accomplished by expressing fluorescently tagged nucleoporins that localize to the nuclear pore complex (NPC) or lamin proteins that underline the inner nuclear membrane [33,51,52,54,70,71]. Live-cell imaging can readily identify single virus docking at the nuclear envelope (NE) based upon the restricted viral motion while colocalizing with an NE marker. Recently, Burdick and coauthors [32] have visualized the interactions between HIV-1 complexes labeled with A3F-YFP and the NE labeled with POM121-mCherry, a nucleoporin which resides in the central channel of the NPC. The authors have demonstrated that a relatively stable (>5 min) association of HIV-1 with the nuclear membrane is dependent on the CA binding to nucleoporin 358 (NUP358). In agreement with the previous results [3,72], depletion of this host factor significantly diminishes the stable NE association events [32], implicating NUP358 in HIV-1 docking at the nuclear pore.

Further support for the role of CA–host factor interactions in HIV-1 docking has been obtained by demonstrating the ability of a small molecule CA-binding inhibitor to displace HIV-1 cores docked at the NE [28]. PF-3450074 (PF-74) binds a pocket formed by an interface between the CA N-terminal and C-terminal domains [73], which overlaps with the binding sites of the cellular proteins, NUP153 and CPSF6, involved in the nuclear import of HIV-1 [74,75]. Treatment of cells with PF-74, which blocks HIV-1 nuclear import [28,71], released the already docked viral complexes into the cytoplasm [28]. This important result implies that the CA–host factor interactions determine the virus docking at the NE.

## 7. Uncoating at the Nuclear Membrane Is a Prerequisite for HIV-1 Nuclear Entry

Live-cell visualization of the HIV-1 nuclear import has been previously achieved by labeling with IN-TC/FlAsh [31] and, more recently, with A3F-YFP or IN-YFP [32]. By co-labeling HIV-1 with CypA-DsRed/CA and INsfGFP and performing three-color live-cell imaging in TZM-bl cells expressing the NE marker EBFP2-LaminB1, we have examined single virus uncoating and nuclear import. In order to adequately resolve processes occurring at different time scales and to minimize photobleaching, the full cell volume has been imaged at different temporal resolutions, from a fraction of a minute to 30 min between image acquisitions. These different image acquisition modes allow one to kinetically resolve individual steps of HIV-1 entry and monitor the infection process in its entirety.

Typically, viral particles exhibit rapid movement in the cytoplasm prior to docking at the nuclear membrane. Single particle tracking reveals restricted virus motion following the INsfGFP co-localization with EBFP2-lamin, which is a manifestation of docking at the NE (Figure 2b–d) [28]. For CypA-DsRed/INsfGFP-labeled viral complexes that subsequently enter the nucleus, docking at the NE is followed by the drop in the CypA-DsRed signal to a background or near-background level, indicating uncoating. After a pronounced lag following the loss of CypA-DsRed, the INsfGFP complexes are quickly transported into the nucleus, on average 1.8 µm from the NE, where they exhibit restricted motion (Figure 2b–d). This docking and nuclear entry dynamic is consistent with the previous studies [31,32]. Importantly, viral cores that dock at the NE, but do not shed a major proportion of CypA-DsRed, fail to enter the nucleus, and are often observed to eventually detach from the NE.

Analysis of the CypA-DsRed intensity of single particles prior to and after docking at the NE has revealed an accelerated loss of this CA marker after docking and showed that this loss was a prerequisite for the nuclear penetration of IN complexes. Interestingly, a fraction of nuclear INsfGFP complexes retained detectable amounts of CypA-DsRed and p24, as determined by immunofluorescence experiments [28]. This finding is in excellent agreement with the low levels of CA associated with the nuclear viral complexes and with vDNA in cell lines and primary human macrophages [32,53,71,76]. Long-term tracking of single INsfGFP complexes that entered the nucleus after CypA-DsRed loss at the NE, showed that the residual CypA-DsRed signal (if any) remains stably associated with the nuclear INsfGFP for several hours. This important result implies that the entire releasable pool of CypA-DsRed is lost at the nuclear pore and that terminal release of CA takes place immediately prior to nuclear import (Figure 2e). Finally, a proportional decrease in the CA/p24 signal, as determined after immunofluorescence labeling, and in CypA-DsRed intensity associated with the viral complexes after nuclear import further supports the notion that loss of CypA-DsRed at the NE is a reliable measure of HIV-1 uncoating.

## 8. HIV-1 Nuclear Import Occurs after a Pronounced Lag Following the Loss of CypA-DsRed/CA at the NE

The docking time of a virus at the NE can be measured as the time interval between arrival/association with the NE and nuclear entry of a single viral complex (Figure 2c). In addition, HIV-1 labeling with CypA-DsRed allows resolving the lag time from docking at the NE to uncoating defined as a terminal loss of the CypA-DsRed signal. In these experiments, single HIV-1 cores uncoat, on average, within ~17 min after docking while the nuclear import occurs after an additional lag of ~20 min after loss of CypA-DsRed. A significant lag between uncoating and nuclear entry suggests the presence of an additional kinetic barrier for nuclear penetration. Importantly, labeling of HIV-1 with CypA-DsRed does not alter the kinetics of nuclear entry or the total time of single virus residence at the NE (docking) prior to nuclear import [28]. The mean docking time at the NE is not significantly affected by knock-out of the target cell CypA [28]. Although somewhat longer docking times in HeLa-derived cells have been observed by Burdick et al. [32], the quantitative difference in the mean docking time may be related to the choice of the NE marker in these two studies. Neither the docking time distribution nor the kinetics of nuclear entry were affected by inhibition of reverse transcription [28,32], implying that the viral DNA synthesis is not strictly required for uncoating that ensures nuclear penetration of RTC/PICs.

Interestingly, the CA N74D mutant that enters the nucleus through an alternative pathway [65,77] also uncoats at the NE, but remains adjacent to the nuclear membrane. The lack of clear nuclear penetration does not allow the measurements of the docking time for this mutant. However, the N74D complexes exhibit a >3-fold slower uncoating after docking at the NE, as compared to the wild-type virus, implicating CA-interacting host factors at the NE in facilitating the accelerated HIV-1 uncoating and nuclear penetration (see below and [28]).

## 9. Visualization of Functionally Relevant Single HIV-1 Entry/Uncoating Events

In addition to the lack of real-time assays for HIV-1 uncoating, a controversy surrounding this process stemmed from the inability to relate the observed readouts to productive infection. In a ground-breaking paper, Mamede and co-authors [29], have pioneered a live-cell imaging approach that relates the early viral fusion/uncoating events to infection. The authors take advantage of the HIV-1 particles labeled with GagiGFP, in which a subset of GFP produced by the Gag-iGFP cleavage appears to be trapped inside a mature viral core [78]. Consequently, the HIV-1 fusion results in a release of the majority of GFP molecules into the cytoplasm, while a subsequent loss of the core integrity is manifested in the second step of GFP release associated with the core-trapped GFP. This assay is thus ideally suited for sensitive detection of the initiation of HIV-1 uncoating. By performing long-term imaging of cells with no more than one virus co-labeled with mCherry-Vpr per cell, the authors have identified the fusion/core-integrity loss events culminating in infection, as determined by the subsequent expression of Gag-iGFP. Importantly, only the 2-step GFP release events resulted in infection, whereas single-step release (presumably corresponding to viruses with defective/unsealed cores) or incomplete GFP release events were not linked to productive entry. These findings imply that: (1) only particles containing a sealed mature core can be infectious; and (2) HIV-1 uncoating, at least the loss of core integrity, is initiated in the cytoplasm at early times after viral fusion. A major limitation of this assay lies in its inability to visualize subsequent steps of uncoating that occur at later times (see also above and [28,32]).

**Figure 3:**
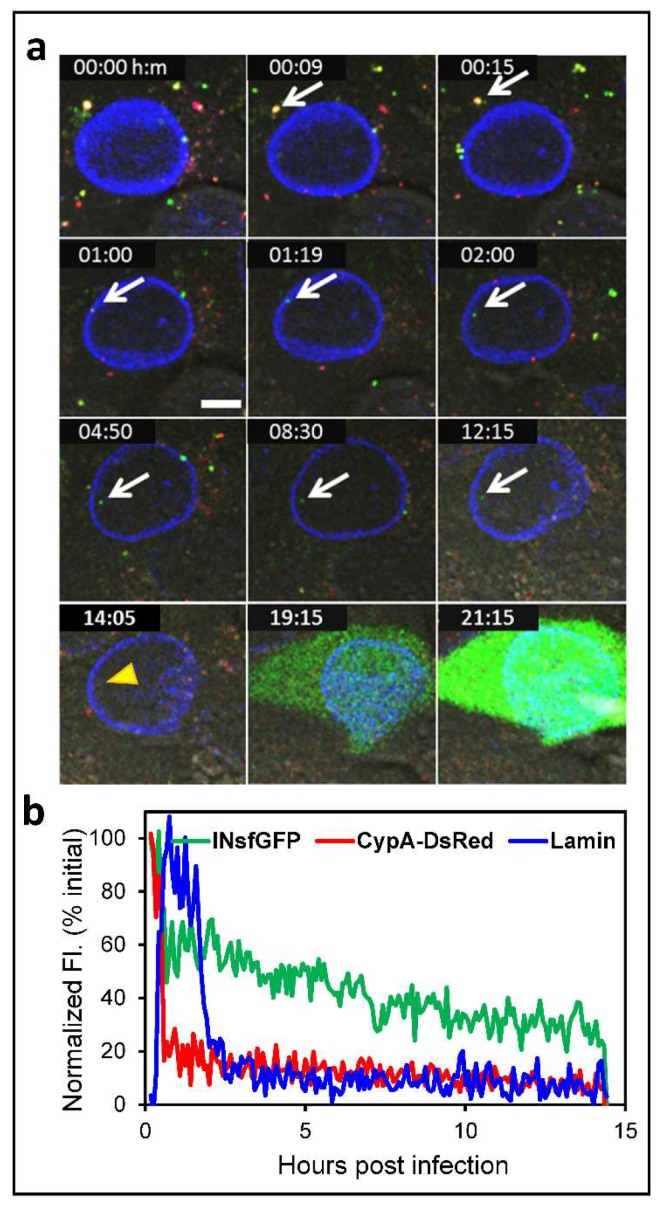
Live cell imaging of single HIV-1 uncoating and nuclear import that culminate in infection. TZM-bl cells expressing EBFP2-Lamin were infected with VSV-G pseudotyped HIV-1 encoding for the eGFP reporter. Viruses were labeled with the INsfGFP and CypA-DsRed markers. Confocal time-lapse images were acquired every 5 min from 0 to 24 h post-infection. (**a**) Time-lapse images of single HIV-1 entry and infection. The arrow marks a single INsfGFP labeled HIV-1 complex in the cytoplasm that docks at the NE, uncoats, and enters the nucleus and disappeared at 14:05 hours post-infection (yellow arrowhead). Loss of INsfGFP signal is followed by expression of eGFP reporter of infection. Scale bar 5 µm. (**b**) Fluorescence intensity traces of the virus in (**a**) that undergoes terminal uncoating after engaging the EBFP2-Lamin labeled NE (manifested in the increase in lamin signal) and enters the nucleus (drop in the lamin signal). Single particle tracking was performed using INsfGFP as reference. The drop in the INsfGFP signal at ~14 h post-infection marks disappearance of the IN spot prior to eGFP expression. Scale bar 5µm.

We have recently performed extended time-lapse imaging of single virus infection using eGFP-encoding HIV-1 pseudoviruses co-labeled with INsfGFP and CypA-DsRed [28]. Live-cell imaging of TZM-bl cell expressing the nuclear membrane marker EBFP2-LaminB1 is performed between 0 and 24 h post-infection. In order to avoid cytotoxicity and to enable sensitive detection of fluorescent nuclear IN complex, images are acquired every 10–30 min, while adequately sampling in the Z-dimension. Analysis of the number of viral complexes in the nucleus shows that, on an average, only ~2% of cell-bound viruses enter the nucleus (Figure 3a), in agreement with Burdick et al. [32]. This imaging strategy has revealed that nearly all cells expressing the eGFP reporter of infection contain at least 1 detectable INsfGFP complex in the nucleus (Figure 3a). The probability of infection strongly correlates with the number of nuclear INsfGFP complexes and poorly correlates with the number of cell-bound viruses, the majority of which are degraded in the cytoplasm after early uncoating [28].

Interestingly, although nuclear IN complexes can usually be tracked for hours, a fraction of these complexes disappears after varied times following the nuclear import (Figure 3a, arrowhead). Most importantly, the disappearance of nuclear INsfGFP complexes was highly predictive of subsequent expression of the eGFP reporter: more than 80% of single nuclear INsfGFP disappearance events culminated in eGFP expression [28]. A similar disappearance of the FlAsh-labeled IN puncta in the nucleus has been previously reported [31] and speculated to represent integration events. We obtained evidence supporting the notion that INsfGFP disappearance corresponds to productive integration. First, the kinetics of nuclear IN disappearance and of the completion of integration, as determined by the time of the integrase inhibitor Raltegravir (RAL) addition experiments, are virtually identical [28]. Second, RAL abrogates the loss of single INsfGFP puncta in the nucleus and thereby increases the total number of nuclear complexes, in full agreement with the previous results obtained by fixed cell imaging [54]. We have therefore examined the upstream trafficking and uncoating steps of single IN complexes that disappeared in the nucleus to determine the location and timing of productive uncoating. Without an exception, productive uncoating occurs after docking at the NE (Figure 3a). Interestingly, the presence of residual CypA-DsRed in viral complexes that were imported into the nucleus did not affect the probability of infection, as determined by analysis of the relationship between the loss of IN complexes and eGFP expression.

For the N74D CA mutant that does not noticeably penetrate into the nucleoplasm, the INsfGFP disappearance after uncoating has also been observed, but these events occurred at the nuclear membrane. Similar to the wild-type CA containing viruses, disappearance of the post-uncoating N74D IN complexes is highly predictive of the subsequent expression of eGFP. This finding implies that, in spite of an apparent co-localization with the nuclear membrane, the N74D complexes shallowly penetrate into the nucleoplasm where they undergo productive integration. This notion is in excellent agreement with different intra-nuclear localization of viral DNA that has been reported for the wild-type and mutant N74D infections [76,79].

In conclusion, terminal loss of CA at the NE is required for HIV-1 nuclear entry (Figure 2e and Figure 3b). This finding suggests that long-lived, gradually uncoating cores are more likely to enter the nucleus and establish infection than the cores undergoing early/abrupt uncoating in the cytoplasm. This result is in line with the observation that early loss of core integrity (second step of GFP release) leads to viral infection reported by Mamede et al. [29]. Note, however, that the overwhelming majority of the two-step GFP release events does not lead to infection [29], perhaps corresponding to the early/abrupt CypA-DsRed loss observed in our recent study [28]. It is therefore possible that early loss of core integrity detected by the second release of GFP and a terminal loss of CypA-DsRed at the NE mark the initial and final steps of the functional uncoating process, respectively. We further conclude that CA–host factor interaction mediates uncoating at the NE, intra-nuclear transport, and potentially determines the HIV-1 integration site preference.

## 10. Summary and Future Perspectives

An increasing number of researchers employ live-cell single virus imaging to gain mechanistic insights into important questions that are difficult to address using more conventional bulk assays. Success of these studies largely depends on developing non-invasive (or minimally invasive) virus labeling approaches. Here, we reviewed two novel labeling strategies developed in our laboratory that allow us to: (1) sensitively detect single HIV-1 fusion and predict the viral protease activity in single particles; and (2) label HIV-1 CA to visualize single particle uncoating in vitro and in living cells. Both these markers can be applied to detect virus fusion and uncoating in several cell-lines and, more importantly, in primary CD4 T cells and macrophages. We also envision that the mCherry-2xCL-eYFPVpr marker will be adaptable to studies of the HIV-1 maturation kinetics, as well as correlative light-electron microscopy (CLEM) studies of post-fusion cores.

Currently, the CypA-DsRed and INsfGFP markers allow linking the uncoating and nuclear entry events to subsequent IN disappearance as a surrogate for productive integration. Further development of novel virus labeling tools holds a great promise for obtaining critical insights into the essential steps of HIV-1 entry en route to integration. A recent report [80] used the Clustered Regularly Interspaced Short Palindromic Repeats (CRISPR) and CRISPR-associated protein 9 (Cas9) methodology to detect the HIV-1 genome in living cells. A combination of this technique with the HIV-1 labeling tools (e.g., IN-sfGFP and CypA-DsRed) will help image the entire single HIV-1 infection process.

Long-term live-cell imaging will be aided by the continuous efforts to develop new genetically encoded and synthetic fluorescent probes. Labeling of viral and cellular proteins with fluorescent tags, such as fluorigen acceptor peptide (FAP), as well as the SNAP and CLIP tags, will afford a great deal of flexibility for multicolor protein labeling that is not achievable with fluorescent proteins. These labeling approaches are compatible with stochastic emission and depletion (STED) imaging, a super-resolution microscopy technique that can be performed in living cells. Further improvements of fluorescence detectors and implementation of light-sheet microscopy (selective plane illumination microscopy (SPIM [81]) will help minimize photobleaching and phototoxicity. These advances will enable more frequent long-term live-cell imaging for longer than 24 h and thus reveal a detailed picture of the entire HIV-1 infectious cycle at a single virus level.
